# Hepatic cavernous hemangioma developed in non-small cell lung cancer patients after receiving Camrelizumab treatment: two case reports

**DOI:** 10.3389/fonc.2023.1221309

**Published:** 2023-08-03

**Authors:** Yonglong Jin, Jinpeng Xu, Dunmin Zhuang, Lina Dong, Yang Sun, Lin Zhao, Wenjing Xiao

**Affiliations:** ^1^ Department of Radiotherapy, Affiliated Hospital of Qingdao University, Qingdao, China; ^2^ School of Public Health, Qingdao University, Qingdao, China

**Keywords:** non-small cell lung cancer, PD-1 inhibitors, Camrelizumab, cavernous hemangioma, case reports

## Abstract

**Purpose:**

To report two cases of hepatic cavernous hemangioma, a rare complication, in patients with locally advanced and advanced non-squamous non-small cell lung cancer (NSCLC) treated with PD-1 inhibitors. Additionally, to share clinical experiences related to the management of this condition.

**Methods:**

Two patients with locally advanced and advanced non-squamous non-small cell lung cancer (NSCLC) were enrolled in our hospital. Following the NCCN guidelines and expert consensus, both patients received standard treatment with Camrelizumab (PD-1 inhibitor). Subsequent abdominal CT scans revealed hepatic focal lesions that did not exhibit typical characteristics of metastatic tumors. Therefore, further systematic investigation was conducted to study the hepatic focal lesions.

**Results:**

(1) Ultrasound-guided percutaneous biopsy confirmed the diagnosis of hepatic cavernous hemangioma. A multidisciplinary consultation concluded that it was an adverse drug reaction to Camrelizumab. (2) Ten-gene testing for both patients did not reveal any driver gene mutations associated with lung cancer. Apart from the occurrence of hepatic cavernous hemangioma, there were no signs of disease progression or worsening. (3) Both patients had resolution of hepatic cavernous hemangioma after switching to alternative PD-1 inhibitors or discontinuing PD-1 inhibitor treatment. One patient experienced hemorrhage related to the hepatic hemangioma, which was managed with hemostasis and symptomatic treatment, resulting in improvement. (4) Clinical outcomes: The first patient achieved a progression-free survival (PFS) of 33 months in first-line treatment and had not reached the PFS endpoint in second-line treatment, with an overall survival exceeding 56 months. The second patient had not reached the PFS endpoint in first-line treatment, with an overall survival exceeding 31 months.

**Conclusion:**

Hepatic cavernous hemangioma is a rare and serious adverse reaction associated with PD-1 inhibitors. Camrelizumab may interact with the PD-1 molecule in a different manner compared to other PD-1 inhibitors, affecting the regulation of the VEGFR/ULBP2 signaling pathway. In future studies, next-generation sequencing may provide detailed molecular pathology information, which could help explain individual differences and provide a basis for the prevention or intervention of hepatic cavernous hemangioma.

## 1 Introduction

Non-Small Cell Lung Cancer (NSCLC) is one of the most common types of lung cancer, accounting for 85-90% of all lung cancer cases (1), and it is currently one of the leading causes of death in both men and women worldwide. Early symptoms of non-small cell lung cancer are often not apparent, and most patients are diagnosed at an advanced stage. According to the data from the American Cancer Society (ACS), approximately 75-80% of NSCLC patients are diagnosed at a locally advanced or advanced stage (2). A large-scale study indicated that the 5-year survival rate for locally advanced NSCLC patients without any treatment is 5%, and even with traditional standardized treatment, the average survival rate is only 25-30% (3, 4).

Immune Checkpoint Inhibitors (ICI) are a novel class of anti-tumor drugs that target the immune system. They work by specifically blocking signals such as PD-1, PD-L1, and CTLA-4, thereby enhancing the body’s ability to recognize and kill tumor cells. Multiple clinical studies, including KEYNOTE, IMpower, and CheckMate trials, have confirmed the anti-tumor effects of PD-1 inhibitors in various solid tumors, particularly when correlated with PD-L1 expression and tumor mutational burden (TMB) (5–8). The National Comprehensive Cancer Network (NCCN) guidelines have upgraded PD-1 inhibitors to category 1A recommendation for second-line treatment in locally advanced or metastatic non-squamous non-small cell lung cancer patients who have not received prior PD-1/PD-L1 inhibitors (9, 10). Camrelizumab, a PD-1 inhibitor, has been approved by the China Food and Drug Administration (CFDA) and widely used in first-line and subsequent-line treatment of locally advanced or advanced non-squamous NSCLC in China, showing favorable outcomes (11, 12). However, ICI therapy is also associated with adverse reactions. Common adverse reactions observed in clinical practice include rash (10-20%), fatigue (10-20%), gastrointestinal reactions (10-20%), elevated liver enzymes (5-10%), pneumonia (2-5%), and thyroid toxicity (1-2%) (13, 14). Reactive skin capillary proliferation is a relatively common adverse reaction of Camrelizumab, mostly presenting as grade 1-2. This adverse reaction generally does not affect patients’ condition or quality of life, and does not require specific treatment. It can resolve on its own after discontinuation of the medication. In addition to the commonly observed adverse reactions, there are rare but potentially life-threatening adverse events (15). Therefore, clinicians using PD-1 inhibitors need to closely monitor and manage adverse reactions in addition to assessing clinical efficacy to prevent serious incidents.

We hereby report two cases of non-squamous non-small cell lung cancer (NSCLC) where rare hepatic cavernous hemangioma developed following second-line treatment with PD-1 inhibitors. We also share our treatment experience.

## 2 Case presentation

### 2.1 Case1

**Figure 1:**
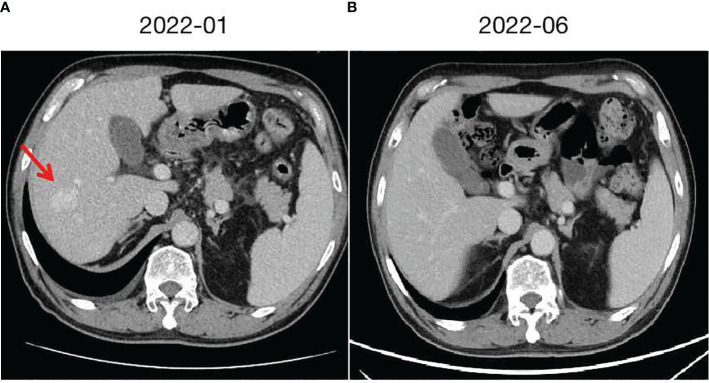
Hepatic cavernous hemangioma and changes. **(A)** taken in January 2022, shows the radiological presentation of a large hepatic cavernous hemangioma on the right lobe. **(B)** taken in June 2022 after switching from Camrelizumab to Tislelizumab, demonstrates the changes observed, with a significant reduction and near disappearance of the hepatic cavernous hemangioma.

In August 2018, an elderly male patient underwent a chest CT scan during a routine check-up, which revealed a 24×25mm lesion in the lower lobe of the left lung. Clinical presentation includes occasional cough that can resolve on its own, without taking any cough suppressant medication. No coughing up phlegm, no significant chest pain, no chest tightness or shortness of breath, no fever or sweating, no abdominal pain, no diarrhea or constipation. Previously in good health. Denies a history of lung diseases such as tuberculosis or pneumonia. Denies a history of infectious diseases such as hepatitis or enteritis. No history of trauma, surgery, or blood transfusion. No history of food or drug allergies. 33-year smoking history, consuming 20 cigarettes per day. Occasional alcohol consumption.Vital signs are stable. Specifically: H 175cm, W 66kg, HR 82/min, BP 126/75mmHg.Laboratory tests: WBC 5.35×10^9^/L (4-10×10^9^/L), RBC 4.24×10^12^/L (4-5.5×10^12^/L), Hb 135g/L(120-160g/L), PLT 125×10^9^/L (100-300×10^9^/L), CEA 71.5ng/ml↑ (<3.4ng/ml), CA24-2 89U/ml↑ (<15U/ml), other tumor markers are within normal range. Enlarged left hilar and mediastinal lymph nodes were also observed, indicating possible metastasis. To determine the nature of the lesion, a CT-guided biopsy of the left lower lobe lesion was performed, and the pathological examination confirmed poorly differentiated adenocarcinoma. The 10-gene panel testing for lung cancer (EGFR, ALK, ROS1, BRAF, KRAS, NRAS, HER2, PIK3CA, RET, MET) showed negative results, and the PD-L1 expression was positive at a rate of 20%. Additional imaging studies were conducted, and no evidence of metastatic lesions was found. The patient was clinically staged as cT1N3M0 IIIB. A multidisciplinary consultation involving thoracic surgery, radiology, pathology, medical oncology, radiation oncology, and respiratory medicine was conducted, and the recommendation was to proceed with concurrent chemoradiotherapy followed by maintenance therapy. From September 2018 to March 2019, the patient underwent 8 cycles of PP regimen chemotherapy (Pemetrexed 500mg/m^2^ IV day 1, Nedaplatin 120mg/m^2^ IV day 1, every 3 weeks) in combination with anti-angiogenic therapy (Endostatin 30mg/day from day 1 to 7, every 3 weeks). During the 3rd to 4th cycle of treatment, concurrent chemoradiotherapy was administered. The specific radiotherapy plan involved 6MV-X rays, delivering a dose of 66Gy to 95% of the planning target volume (PTV) over 33 fractions for a duration of 6 to 7 weeks. The treatment toxicity was manageable. After completion of the chemoradiotherapy, the treatment response was evaluated according to the RECIST v1.1 criteria, which indicated a Partial Remission (PR). At that time in China, PD-1 inhibitors had not yet obtained indications for maintenance therapy in stage IIIB non-small cell lung cancer. Results from the Beyond study, focusing on the Chinese population, demonstrated that during the maintenance phase of non-small cell lung cancer treatment, the combination of pemetrexed with anti-angiogenic therapy improved Overall Response Rate (ORR) and Time to Progression (TTP). Therefore, subsequently, pemetrexed in combination with endostatin was administered as maintenance therapy (Pemetrexed 500mg/m^2^ IV day 1, Endostatin 30mg/day from day 1 to 7, every 3 weeks).In May 2021, a follow-up chest CT revealed an increased size of the residual lesion in the left lung accompanied by carcinomatous lymphangitis, and the treatment response was evaluated as Progressive Disease (PD), suggesting possible resistance. From June 2021 to December 2021, the patient received a second-line treatment regimen (Camrelizumab <PD-1 inhibitor> 200mg IV day 1, Bevacizumab 500mg IV day 1, every 3 weeks) for a total of 8 cycles. The treatment response assessment after the 2nd and 4th cycles indicated Partial Remission (PR), while the assessment after the 6th and 8th cycles showed Stable Disease (SD).In January 2022, a follow-up examination revealed a nodular lesion in the right liver (**Figure 1A**). Ultrasound-guided biopsy was performed, and the pathological result indicated hepatic cavernous hemangioma. Following the biopsy, there was an occurrence of intraperitoneal bleeding, resulting in the drainage of 3500ml of hemorrhagic ascites. Cell pathology examination did not reveal malignant tumor cells, and the patient’s condition improved after receiving symptomatic treatment for hemostasis. Considering the change in the patient’s condition, a multidisciplinary consultation was conducted again, and it was speculated that the occurrence of hepatic cavernous hemangioma could not be ruled out as an adverse drug reaction to Camrelizumab, with the possibility that Bevacizumab might exacerbate the risk of bleeding. Based on the patient’s condition and the consultation results, after a two-month period of recuperation, the patient commenced a new treatment regimen in April 2022, which involved switching the immune checkpoint inhibitor and continuing with Bevacizumab. The immune checkpoint inhibitor was changed from Camrelizumab to Tislelizumab(200mg ivd d1, q3w), while Bevacizumab treatment remained the same. Regular follow-up examinations every two months indicated sustained stable disease in the primary lesion, and the hepatic cavernous hemangioma disappeared (**Figure 1B**). These results suggest a close association between the occurrence of hepatic cavernous hemangioma and Camrelizumab, and that there is a risk of intraperitoneal bleeding with the combination of anti-angiogenic therapy. However, the bleeding risk was reversible after discontinuation of the medication.

### 2.2 Case2

**Figure 2:**
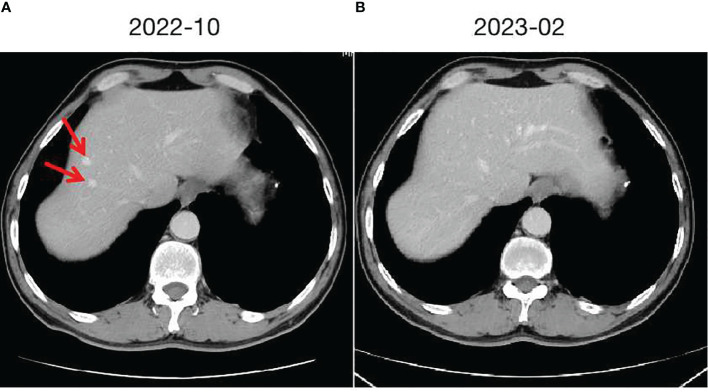
Hepatic cavernous hemangioma and changes. **(A)** taken in October 2022, shows the initial imaging findings of two hepatic cavernous hemangiomas in the left liver. **(B)** taken in February 2023, demonstrates the changes following discontinuation of Camrelizumab, with the hepatic cavernous hemangioma almost disappearing.

In September 2020, a middle-aged male presented to the hospital with dizziness and headaches. Brain MRI revealed an abnormal mass in the brain, suggestive of a metastatic tumor. Apart from neurological symptoms, there is mild cough without sputum, no chest tightness or chest pain, no fever or sweating, no diarrhea or constipation. The patient has a history of hypertension for 10 years. The highest recorded blood pressure was 175/105mmHg. Currently, the patient is taking oral antihypertensive medication (Valsartan capsules, 80mg/d), and the blood pressure is controlled within the range of 130-140/90-100mmHg. No cerebrovascular diseases or coronary artery disease. The patient denies any infectious diseases such as hepatitis or enteritis. There is no history of trauma, surgery, or blood transfusion. No known food or drug allergies. The patient has a smoking history of 35 years, consuming 10 cigarettes per day. Occasional alcohol consumption.Vital signs are stable. Specifically:H 177cm, W 60kg, HR 91/min, BP 137/95mmHg.Laboratory tests: WBC 7.64×10^9^/L (4-10×10^9^/L), RBC 5.17×10^12^/L (4-5.5×10^12^/L), Hb 128g/L (120-160g/L), PLT 232×10^9^/L (100-300×10^9^/L), CEA 103.6ng/ml↑ (<3.4ng/ml), CA24-2 63U/ml↑ (<15U/ml), other tumor markers are within normal range. Further evaluation with whole-body PET/CT indicated a lesion in the left lung measuring 40×35mm, accompanied by lymph node and mediastinal metastasis, as well as multiple brain metastases. A CT-guided biopsy was performed on the lesion in the left lung, and the pathological analysis indicated poorly differentiated adenocarcinoma. A 10-gene test for lung cancer revealed a KRAS missense mutation (G13D, 13.91%), while no mutations were found in the remaining genes (EGFR, ALK, ROS1, BRAF, NRAS, HER2, PIK3CA, RET, MET). The PD-L1 positivity rate was 10%.The clinical stage is cT2N2M1, stage IV. A multidisciplinary consultation within the hospital was conducted, considering the patient’s current multiple brain metastases and significant symptoms. It was recommended to prioritize whole-brain radiation therapy (WBRT) and symptomatic treatments such as intracranial pressure reduction and nutritional support. After symptom control, systemic treatment and radiation therapy for the primary lesion were suggested. Consequently, from October 2020 to November 2020, the patient received WBRT using 6MV X-rays, delivering a dose of 30Gy/10f/2w to 95% of the planning target volume (PTV). During this period, treatment for dehydration and intracranial pressure reduction was administered. Following the completion of radiation therapy, the patient experienced a significant improvement in symptoms, including reduced dizziness and headaches. The ECOG PS improved from a score of 3 to 1.Although the patient was initially diagnosed with stage IV disease, distant metastases were limited to the intracranial region. After receiving cranial radiation therapy and supportive care, the intracranial lesions were well controlled, and the patient’s general condition remained good. The patient expressed a strong willingness for treatment. Taking reference from the KEYNOTE189 and CAMEL studies, after discussing with the patient, a systemic treatment regimen comprising platinum-based doublet chemotherapy in combination with PD-1 inhibitor was administered. From December 2020 to June 2021, the patient received 8 cycles of the PP regimen chemotherapy (Pemetrexed 500mg/m² iv day 1, Carboplatin AUC5 iv day 1, every 3 weeks) combined with PD-1 inhibitor therapy (Camrelizumab 200mg, every 3 weeks). During the second to fourth cycles of treatment, concurrent radiotherapy was administered. The specific radiotherapy plan consisted of 6MV X-rays, delivering a dose of 60Gy/30f/6w to 95% of the planning target volume (PTV), and the treatment toxicity was tolerable. Following the completion of concurrent chemoradiotherapy, the therapeutic evaluation of the primary lesion in the chest and intracranial lesions, according to RECIST v1.1 criteria, showed a partial response (PR). Maintenance treatment was initiated in July 2021, with the following regimen: Pemetrexed 500mg/m² iv day 1, Bevacizumab 500mg iv day 1, and Camrelizumab 200mg every 3 weeks. Regular follow-up examinations were conducted every 2 months, and the therapeutic evaluation remained stable disease (SD). In October 2022, a liver nodule was discovered in the left liver lobe on abdominal CT (**Figure 2A**). A further ultrasound-guided biopsy was performed, and the pathological results confirmed hepatic cavernous hemangioma. Similar to the previous patient, a multidisciplinary consultation was conducted, and it was determined that the liver nodule was likely a drug reaction to Camrelizumab. Therefore, Camrelizumab was discontinued starting from November 2022, and maintenance treatment with Pemetrexed and Bevacizumab was initiated using the same dosage and cycle as before. Subsequently, the hepatic cavernous hemangioma disappeared (**Figure 2B**), and no further drug-related adverse reactions occurred. The clinical response continued to be evaluated as stable disease (SD).

## 3 Discussions

According to the latest statistics, lung cancer remains the most common and deadliest malignant tumor worldwide, with non-small cell lung cancer (NSCLC) accounting for over 80% of lung cancer cases. With the advancement of diagnostic and therapeutic technologies, the treatment of advanced NSCLC has gradually transitioned towards a personalized approach. Based on clinical diagnosis and molecular subtyping, targeted therapy with small molecule inhibitors is employed for patients with driver gene mutations, while immune checkpoint inhibitors (ICIs) targeting PD-1/PD-L1 are used for patients without driver gene mutations. Based on the KEYNOTE-189/KEYNOTE-407 studies, the U.S. FDA has approved pembrolizumab in combination with pemetrexed/platinum-based chemotherapy for first-line treatment of stage IV non-squamous/non-small cell lung cancer (NSCLC) patients who are negative for EGFR/ALK mutations (16). In China, based on the CameL and Rational series studies, the National Medical Products Administration (NMPA) has approved the use of Camrelizumab or Tislelizumab in combination with Pemetrexed and Carboplatin for first-line treatment of unresectable locally advanced or metastatic non-squamous non-small cell lung cancer (NSCLC) patients who are negative for EGFR/ALK mutations (17). Additionally, the IMpower150 study has demonstrated that the quadruple combination of immune checkpoint inhibitors (ICI), Platinum-based doublet chemotherapy, and anti-angiogenic therapy significantly improves the overall survival of stage IV non-squamous NSCLC patients who are negative for EGFR/ALK mutations compared to chemotherapy alone (OS: 19.2 months vs 14.7 months; HR=0.78, 95% CI: 0.64-0.96) (18). In other words, the combination of immune checkpoint inhibitors (ICI) with other treatment modalities has become a recommended first-line standard treatment regimen in the NCCN guidelines for advanced driver gene-negative non-small cell lung cancer (NSCLC) with PD-L1 expression level less than 50%.

**Figure 3:**
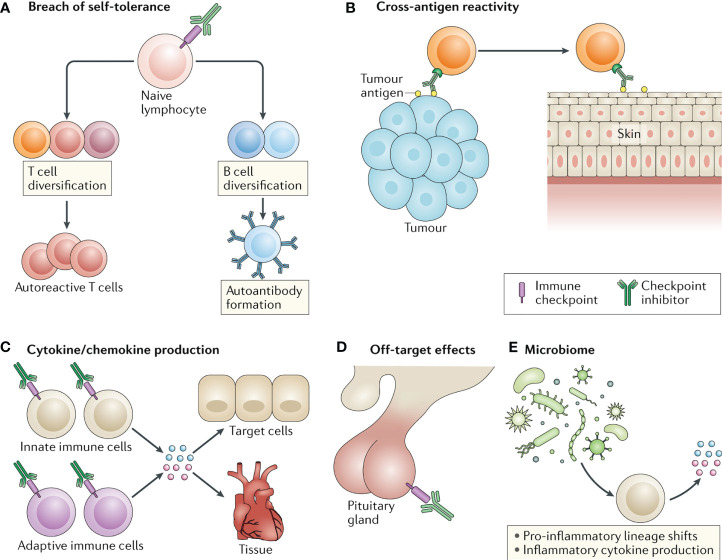
Four common molecular mechanisms underlying the development of irAEs induced by ICIs. (A. disruption of self-immune tolerance; B. cross-reactivity; C. release of cytokines; D. off-target effects).

However, the occurrence of Immune-Related Adverse Events (irAEs) cannot be ignored. These events can affect almost all organ systems, limiting the clinical benefits of the drugs and, in severe cases, even endangering the patient’s life. Statistical results indicate that approximately 40% of tumor patients treated with ICIs will experience various degrees of irAEs, including rash, interstitial pneumonia, enteritis, hepatitis, thyroiditis, and others (19–21). Among them, irAEs affecting the skin, gastrointestinal tract, endocrine system, lungs, and musculoskeletal system are relatively common, while irAEs involving the cardiovascular system, blood, kidneys, nerves, and eyes are less common. Currently, there have been no reported cases of hepatic hemangiomas associated with irAEs. The pathogenesis of irAEs is currently believed to involve activated T cells not only attacking tumor cells but also causing self-damage through mechanisms such as disrupting self-immune tolerance, cross-reactivity, releasing large amounts of cytokines, off-target effects, and interactions with the microbiota (**Figure 3**) (22). Early recognition and proper management of irAEs play a crucial role in maximizing the immunotherapeutic anti-tumor effect and improving patient outcomes. Several authoritative organizations, including the European Society for Medical Oncology (ESMO), the Society for Immunotherapy of Cancer (SITC), the American Society of Clinical Oncology (ASCO), and the Chinese Society of Clinical Oncology (CSCO), emphasize the importance of early diagnosis and appropriate management of irAEs. This includes proactive prevention, baseline assessment, early detection, timely treatment, and dynamic monitoring, with each step deserving sufficient attention.

This study reports on two clinical cases, one with locally advanced non-squamous non-small cell lung cancer (NSCLC) and the other with advanced non-squamous NSCLC. Genetic testing of 10 lung cancer genes indicated no driver gene mutations or indications for targeted therapy. To improve overall survival (OS), progression-free survival (PFS), and quality of life (QOL) for the patients, platinum-based doublet chemotherapy was combined with Camrelizumab (PD-1 inhibitor) and anti-angiogenic targeted therapy, taking into account the patients’ Eastern Cooperative Oncology Group Performance Status (ECOG PS) and treatment guidelines. Concurrent with systemic therapy, local radiotherapy was administered. Both patients achieved favorable clinical treatment outcomes. However, during the regular follow-up examinations in the later stages of treatment, abdominal CT revealed a liver mass, and the pathological results of the biopsy indicated hepatic hemangioma. Additionally, elevated transaminase levels (hepatotoxicity) are one of the more common adverse reactions associated with ICIs. However, the occurrence of hepatic hemangioma can exacerbate hepatotoxicity, leading to more complex and severe hepatic pathophysiological changes, such as significant bleeding, ascites, hypoalbuminemia, and so on, which can be life-threatening. This is an unusual irAE that cannot be explained by the previously known pathogenic mechanisms. While providing symptomatic treatment to the patients, a review of the latest literature did not yield any relevant research content. The main causes of hepatic cavernous hemangioma include congenital developmental abnormalities and hormonal stimulation. Congenital developmental abnormalities are caused by abnormal proliferation of endothelial cells in the peripheral blood vessels of the liver during embryonic development, resulting in the formation of hepatic hemangiomas. Hormonal stimulation factors include female adolescence, pregnancy, oral contraceptives, and hormone therapy involving estrogen and progesterone. These hormonal fluctuations in females can accelerate the growth rate of the vascular tumor, hence 80% of the affected individuals are women. Some scholars also believe that deformation of the capillary tissue occurs after infection, leading to the formation of cavernous dilatation in the blood vessels (23, 24).Both patients in this study were male. Pre-treatment abdominal CT scans did not reveal any abnormalities, and they did not use medications containing female hormones. They denied any previous history of intrahepatic infection symptoms, thereby excluding other high-risk factors for the development of cavernous hemangiomas. Through literature research, we have learned that reactive capillary proliferation is a specific adverse reaction associated with the use of Camrelizumab. When Camrelizumab triggers excessive activation of the immune function, it disrupts the dynamic balance of vascular growth factors within the skin tissue, leading to reactive capillary proliferation (25).In this study, both patients developed hepatic vascular tumors detected by abdominal CT after receiving Camrelizumab, and subsequent histological examination confirmed the presence of cavernous hemangiomas. Considering the pathogenesis of hepatic cavernous hemangioma and the treatment history of the two patients, the multidisciplinary consultation concluded that Camrelizumab was the cause of the hepatic cavernous hemangiomas. It was clear that immediate discontinuation of Camrelizumab should be implemented. Upon discontinuation of Camrelizumab or its replacement with Tislelizumab for continued anti-tumor treatment, it was observed that the hepatic hemangioma in the patients gradually reduced in size, and after a 3-month cessation of Camrelizumab, it had essentially disappeared. This result confirms our speculation that Camrelizumab can reversibly induce hepatic cavernous hemangiomas.

For different PD-1 inhibitors, they bind to different sites on the PD-1 molecule, which may be the basis for the differences in adverse reactions observed among different PD-1 inhibitors. To understand the design differences between Camrelizumab and other PD-1 inhibitors, further analysis of their molecular structures and pharmacological characteristics was conducted. Camrelizumab is a selective, humanized, high-affinity IgG4 monoclonal antibody that binds to the surface of immune cells targeting PD-1.By blocking PD-L1 on the surface of malignant tumor cells, it relieves the immune suppression mediated by the PD-1 pathway, thereby restoring the ability of the body’s immune system to monitor and eliminate tumor cells. This has resulted, it was found that Camrelizumab can bind highly specifically to the C, C’, and FG ends of the PD-1 molecule, enabling it to exhibit high affinity and occupancy for the PD-1 receptor. This allows Camrelizumab to more effectively block the PD-1/PD-L1 pathway and promote the release of IFNγ by T cells (26). Camrelizumab exhibits potent anti-tumor activity with an IC50 of 0.7 nmol/L and an EC50 of 0.38 nnmol/L when binding to PD-1.Tislelizumab, another PD-1 inhibitor approved by the CFDA, is widely used in the treatment of common malignancies. After the first patient developed hepatic cavernous hemangioma associated with Camrelizumab, the PD-1 inhibitor was switched to Tislelizumab, resulting in gradual improvement and recovery of the hepatic cavernous hemangioma. Therefore, when comparing the two, it was found that in the molecular structure of Tislelizumab, all three CDRs of the VL domain and two CDRs (CDR2 and CDR3) of the VH domain are involved in extensive binding to PD-1. Following genetic engineering modifications in the Fc segment, Tislelizumab can significantly reduce binding to Fc receptors in macrophages (27). Whether these structural differences are related to the occurrence of hepatic hemangioma has not been addressed in relevant research studies.

The adverse reactions observed with Camrelizumab are mostly grade 1-2. Among them, reactive capillary proliferation in the skin is a relatively specific and common adverse reaction, with an incidence rate of 77-80%. The earliest and most common sites of occurrence are the face, scalp, neck, and upper chest wall, followed by the abdominal wall, back, and extremities. Morphologically, the most common presentation is a “red mole-like” or “pearl-like” appearance on the skin, which is prone to ulceration and bleeding. The specific pathogenic mechanism is still unclear, but some studies suggest that it may be due to the excessive activation of immune function by Camrelizumab, leading to the upregulation of vascular endothelial growth factor A, which disrupts the dynamic balance between pro-angiogenic factors and anti-angiogenic factors in the skin tissue. These pathological changes promote the proliferation of local capillary endothelial cells and represent an immune-mediated reactive response (28, 29). Teng et al. reported that Camrelizumab can affect the redistribution of blood vessels through the modulation of VEGF-related mechanisms (30). Furthermore, William et al. suggested that PD-1 inhibitors may induce abnormal capillary proliferation in the body through the VEGFR/ULBP2 signaling pathway (31). In the management of hemangiomas, Xue et al. reported that for superficial localized hemangiomas, most cases show spontaneous resolution after discontinuation of medication (32). Zhou et al. reported that oral mucosal hemangiomas can have a favorable prognosis when treated with local surgical excision within one year (33). Li et al. found that the occurrence and progression of cutaneous hemangiomas can be inhibited by the use of VEGF inhibitor Apatinib (34). These studies indicate that Camrelizumab-induced hemangiomas are closely associated with the VEGF signaling pathway in the body and, in most cases, are reversible with improvement observed after discontinuation of the medication or local intervention. Currently, there have been no reported cases of Camrelizumab-induced hepatic hemangiomas. The two cases of hepatic cavernous hemangioma observed in this study are relatively rare sites of occurrence for Camrelizumab and have more severe clinical manifestations. However, with timely discontinuation of the medication and symptomatic management, both patients showed improvement. Additionally, most cases of reactive capillary proliferation associated with Camrelizumab appear within 2 to 4 weeks after the initial dose and, as the number of doses increases, nodules may gradually enlarge and multiply. It is noteworthy that the occurrence of hepatic cavernous hemangioma in these two patients occurred relatively late, and there was a significant difference between the two cases (6 months vs. 22 months). Currently, no relevant studies have reported individual differences in the timing of occurrence. Considering the pharmacokinetics, mechanism of action, and clinical characteristics of the patients described earlier, we believe that the following two factors may be related (1). It is related to tumor burden and PD-L1 expression levels. Firstly, compared to other patients, the adverse reactions appeared later in these two patients. This may be because hepatic cavernous hemangiomas occur in visceral organs, unlike reactive proliferation in the skin, which is more easily detected early on. Additionally, these processes develop more slowly, and imaging examinations may not easily detect them until nodules of a certain size have formed. Therefore, there is a significant delay in the timing of detection. Secondly, the occurrence of hepatic cavernous hemangiomas differs between these two patients. Let’s review the two patients: The first patient experienced recurrence and metastasis after first-line treatment and received Camrelizumab, with a higher tumor burden and higher PD-L1 expression (20%).The second patient received Camrelizumab during the maintenance phase after first-line treatment, with a smaller tumor burden and relatively low PD-L1 expression (10%). In terms of tumor cell quantity and target expression on tumor surface, the first patient had a more abundant profile compared to the second patient, resulting in greater clinical benefit from the treatment. As the use of immunotherapy becomes more widespread, it is increasingly recognized that treatment efficacy is closely related to adverse reactions. Aso et al. analyzed clinical data from 155 patients with advanced non-small cell lung cancer treated with Nivolumab and Pembrolizumab monotherapy and found that patients with skin adverse reactions (51 cases) had a higher objective response rate (57% vs. 19%, *P*<0.01) and longer progression-free survival (12.9 months vs. 3.5 months, *P*<0.01) (35). This indicates that patients with skin adverse reactions derived greater clinical benefits from tumor treatment. Huang et al. conducted a study on 228 patients with advanced or metastatic esophageal squamous cell carcinoma treated with Camrelizumab and found that the median overall survival of patients who developed reactive capillary proliferation was 10.1 months, compared to only 2.5 months for those who did not develop it (36). Recent studies have also shown that reactive capillary proliferation can serve as a clinical indicator for predicting the efficacy of Camrelizumab monotherapy. Therefore, it can be understood why the first patient had more prominent drug-related adverse reactions and an earlier onset compared to the second patient (2). It is related to genotype. In addition to tumor burden and PD-L1 factors, we also believe that individual genotypes play a role. Compared to traditional treatments such as chemotherapy and radiotherapy, immune checkpoint inhibitors are still in the early stages, and many mechanisms and adverse reactions are not yet fully understood. Some individuals may experience synergistic effects and achieve remarkable treatment outcomes, while others may have to discontinue treatment or even experience fatalities due to adverse reactions. These outcomes cannot be well explained by the currently available clinical indicators. Perhaps in the future, with more extensive and precise genetic testing, we will be able to elucidate the reasons for individual differences. In addition to timely management and discontinuation of the medication, prevention and early intervention are particularly important. Referring to the expert consensus published by the American NCCN in CTCAE v5.0 and the “Guidelines for the Management of Immune Checkpoint Inhibitor-Related Toxicities (2021)” published by CSCO, adverse drug reactions should be managed in graded levels (such as bleeding, infection, nodules) and on a long-term basis (37). For Camrelizumab, in addition to baseline assessments, regular skin and mucosal examinations are helpful in early detection and intervention of capillary proliferation and cavernous hemangiomas. For patients who have already developed reactive capillary proliferation, especially when it occurs on the facial region and may lead to negative emotions such as tension, anxiety, and depression, healthcare providers conducting regular health education, psychological counseling, and mental support will help build patient confidence and promote successful completion of treatment.

In this study, we aim to report two rare cases of hepatic hemangiomas and explore the potential underlying mechanisms, in order to provide insights into the prevention and early management of PD-1 inhibitor-induced hepatic hemangiomas. In our previous study (15), we utilized next-generation sequencing analysis on a large cohort and identified co-mutations in CDKN2 and PDL2. We observed that patients with these co-mutations experienced a cytokine storm upon PD-1 inhibitor treatment, leading to severe and potentially fatal adverse reactions. This suggests that, in addition to conventional indicators such as tumor biomarkers, PD-L1 expression, tumor mutational burden (TMB), and microsatellite instability (MSI), next-generation sequencing methods hold promise for providing more robust evidence in rare drug adverse reactions and individual differences. Unfortunately, due to personal reasons, only routine panel testing for the 10 common genes in lung cancer was performed in the two cases reported in this study. Comprehensive NGS was not conducted, thus limiting the acquisition of precise molecular pathological information. Therefore, further exploration of new treatment strategies is necessary to improve the survival of advanced non-small cell lung cancer (NSCLC). This study acknowledges the challenges and issues faced, and future work will continue to focus on this problem, aiming to explore molecular mechanisms and clinical approaches.

## Data availability statement

The original contributions presented in the study are included in the article/supplementary material. Further inquiries can be directed to the corresponding author.

## Ethics statement

Written informed consent was obtained from the individual(s) for the publication of any potentially identifiable images or data included in this article.

## Author contributions

WX designed this project. YJ wrote this paper. JX, DZ, LD, YS, and LZ jointly collected clinical data and image. All authors contributed to the article and approved the submitted version.
